# Modulation of estrogen related receptor alpha activity by the kinesin KIF17

**DOI:** 10.18632/oncotarget.18104

**Published:** 2017-05-23

**Authors:** AM Pramodh Bandara Seneviratne, Zeynep Turan, Aurelie Hermant, Patrick Lecine, William O. Smith, Jean-Paul Borg, Fanny Jaulin, Geri Kreitzer

**Affiliations:** ^1^ Department of Molecular, Cellular & Biomedical Sciences, The City University of New York School of Medicine, New York, NY, USA; ^2^ Department of Cell and Developmental Biology, Weill Medical College, Cornell University, New York, NY, USA; ^3^ Centre de Recherche en Cancérologie de Marseille, Aix Marseille Univ UM105, Institut Paoli-Calmettes, UMR7258 CNRS, U1068 INSERM, Cell Polarity, Cell Signalling and Cancer, Equipe labellisée Ligue Contre le Cancer, Marseille, France; ^4^ The City University of New York School of Medicine, New York, NY, USA; ^5^ Gustave Roussy Institute, Villejuif, France; ^6^ BIOASTER, Tony Garnier, Lyon, France; ^7^ California Institute of Technology, Pasadena, CA, USA

**Keywords:** kinesin, estrogen related receptor alpha, transcription, breast cancer, epithelial

## Abstract

Estrogen-related receptor alpha (ERR1) is an orphan nuclear receptor that can bind transcriptional co-activators constitutively. ERR1 expression correlates with poor patient outcomes in breast cancer, heightening interest in this nuclear receptor as a therapeutic target. Because ERR1 has no known regulatory ligand, a major challenge in targeting its activity is to find cellular or synthetic modulators of its function. We identified an interaction between ERR1 and KIF17, a kinesin-2 family microtubule motor, in a yeast-2-hybrid screen. We confirmed the interaction using *in vitro* biochemical assays and determined that binding is mediated by the ERR1 ligand-binding/AF2 domain and the KIF17 C-terminal tail. Expression of KIF17 tail domain in either ER-negative or ER-positive breast cancer epithelial cells attenuated nuclear accumulation of newly synthesized ERR1 and inhibited ERR1 transcriptional activity. Conversely, ERR1 transcriptional activity was elevated significantly in KIF17 knock-out cells. Sequence analysis of the KIF17 tail domain revealed it contains a nuclear receptor box with a conserved LXXLL motif found in transcriptional co-activators. Expression of a 12 amino-acid peptide containing this motif was sufficient to inhibit ERR1 transcriptional activity and cell invasion, while deletion of this region from the KIF17 tail resulted in increased ERR1 activity. Together, these data suggest KIF17 modifies ERR1 function by two possible, non-exclusive mechanisms: (i) by regulating nuclear-cytoplasmic distribution or (ii) by competing with transcriptional co-activators for binding to ERR1. Thus targeting the ERR1-KIF17 interaction has potential as a novel strategy for treating breast cancer.

## INTRODUCTION

Estrogen related receptor alpha (ERR1) is an orphan nuclear receptor that regulates genes involved in adaptive energy and lipid metabolism, mitochondrial biogenesis, osteogenesis, thermogenesis and ion homeostasis [1, 2]. ERR1 regulates transcription of its gene targets by binding to specific ERR response elements (ERREs) [3–5]. In addition, ERR1 can activate transcription of estrogen receptor alpha (ER) target genes through multiple, imperfect estrogen receptor response element (ERE) half-sites, although this occurs primarily in cells lacking ER [4, 6–10]. ERR1 has a ligand binding pocket, but no natural ligand has been identified to date and ERR1 can bind its coactivators constitutively [9, 11]. However, ERR1 function can be modified in response to signaling through EGF, ErbB2 and IGF-1R, which affect its binding to co-activators and repressors, its transcriptional activity, and its targeting for degradation [12–14]. ERR1 activity may also be regulated by competition with ER for binding to transcriptional co-factors, as it is known to modulate responses to estrogen by competing with ER for binding to EREs on target DNA and to regulatory co-activators or co-repressors [15–17].

ERR1 expression correlates with poor prognoses in breast cancer, particularly in patients with ER-negative, ER-negative/ErbB2-positive, and triple-negative tumors. As such, it has gained attention as a potent factor and possible therapeutic target in treating aggressive disease [18]. Small molecule, selective, inverse-agonists occupy the ERR1 ligand binding domain and induce a conformational change in ERR1 that inhibits binding of ERR1 co-activators, leading ultimately to degradation of ERR1 by the proteasome [19–22]. Importantly, ERR1 inverse agonists are effective in inhibiting orthotopic breast tumor growth in murine models [23, 24]. To date however, there is no evidence that these agents are in clinical trial - perhaps because they would be predicted to induce systemic toxicity in view of ERR1's regulation of genes in multiple essential pathways. Consequently, a key step in development of clinically viable anti-ERR1 therapies is to identify cellular or synthetic modulators that affect only subsets of ERR1-regulated genes that are altered in the disease state.

We identified ERR1 as a binding partner of the kinesin-2 family motor protein KIF17. Kinesins are microtubule (MT) stimulated ATPases that transport a variety of soluble and membrane-bound cargoes within the cell [25, 26]. In humans, the kinesin family is comprised of over 40 related proteins that share structural and functional motifs. Kinesin motor domains are highly conserved and bind MTs and ATP. Kinesin tail domains interact with numerous cellular cargoes but are highly divergent, contributing in large part to the uniqueness between family members. Kinesins are known to participate in regulation of transcriptional signaling [27–30], but their potential to modulate nuclear receptor activity directly in breast cancer has not been investigated. The studies described here show that in ER-positive and ER-negative breast cancer cells, ERR1 localization and activity can be regulated by KIF17. Notably, a 12 amino acid peptide within the KIF17 tail is necessary and sufficient to inhibit ERR1 function. This peptide contains a conserved LXXLL nuclear receptor box motif (NR box) found in nuclear receptor co-activators [31–33]. The LXXLL co-activator motif is sufficient for nuclear receptor (NR) binding. However, flanking amino acids influence which of the many co-activators bind NRs (e.g. in response to signaling); this affects expression of distinct gene targets and likely downstream cellular pathways [31–33]. The NR box in KIF17 shares features with a subset of NR co-activators [31], suggesting that a KIF17 peptide containing this NR box could be exploited to inhibit a subset of ERR1-responsive genes. This could lead to development of new therapeutic strategies targeting ERR1 in breast and potentially other steroidogenic and non-steroidogenic cancers.

## RESULTS

### ERR1 interacts with KIF17

**Figure 1:**
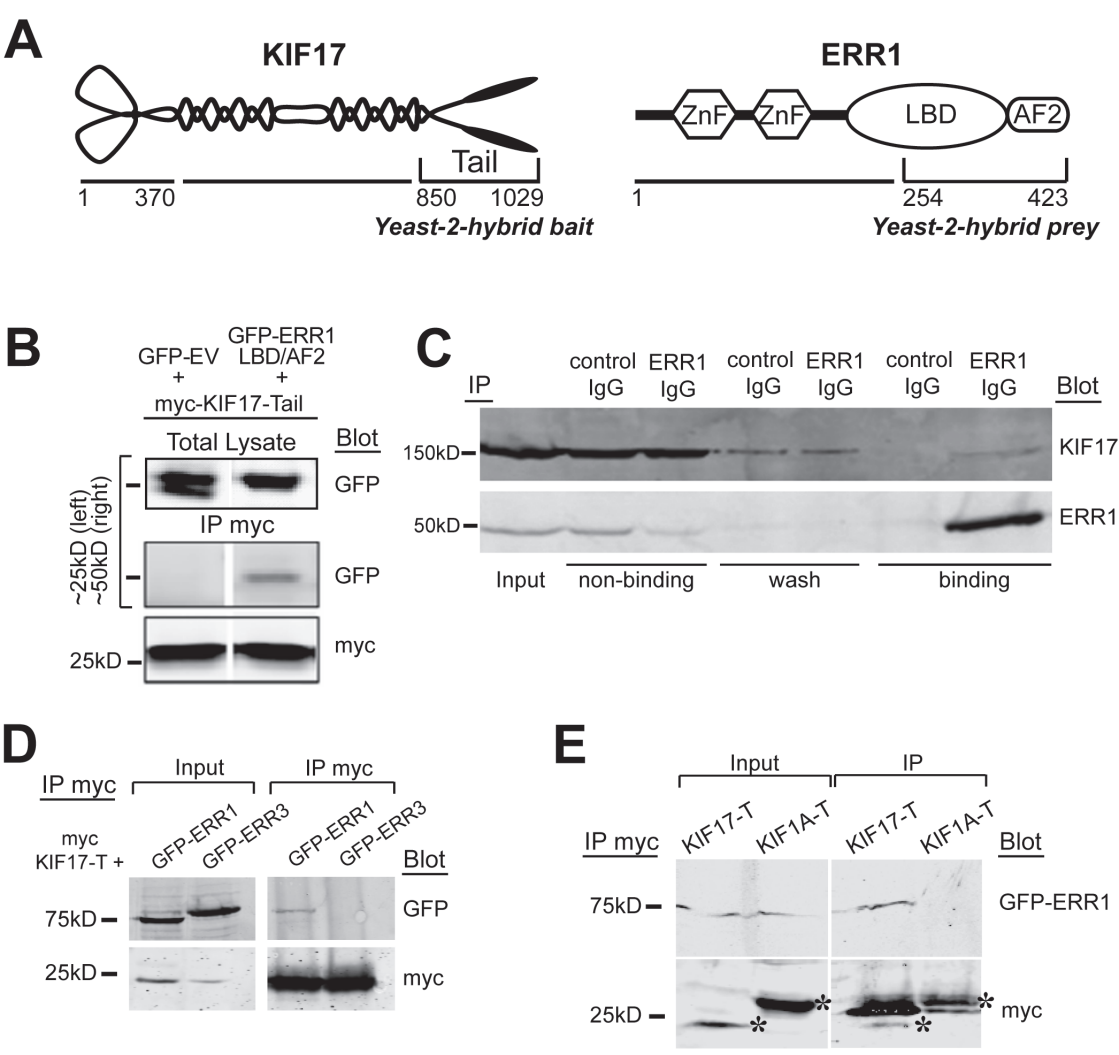
KIF17 interacts selectively with ERR1 **A.** Schematic diagrams of KIF17 and ERR1 and their interacting domains identified in the yeast-2-hybrid screen. **B.** Co-immunoprecipitation of GFP-ERR1 LB/AF2 domain with myc-KIF17-tail in HEK293T cells. Upper left lane shows the ~25kDa marker region where GFP migrates; upper right shows marker at 50kDa for GFP-ERR1-LBD/AF2 migration. **C.** Co-immunoprecipitation of endogenous KIF17 and ERR1 from MCF7 cells. **D.** Co-immunoprecipitation analysis of myc-KIF17-T and full-length GFP-ERR1 or GFP-ERR3 expressed in HEK-293T cells. Note that immunoprecipitated GFP-ERR1 routinely migrates more slowly than the majority of total GFP-ERR1 detected in input lanes. **E.** Co-immunoprecipitation analysis of full-length GFP-ERR1 and myc-KIF17-T or myc-KIF1A-T expressed in HEK-293T cells. Inputs in D and E represents 10% of total lysate from HEK293T cells expressing the indicated constructs. Asterisks in E show myc-tagged kinesin tails. Additional bands in the IP are IgG light chains.

We identified ERR1 as a binding partner of the homodimeric kinesin-2 family motor KIF17 in a yeast-2-hybrid screen of normal epithelial cells using the KIF17 C-terminal tail domain as bait (KIF17-T, Figure 1A). Yeast-2-hybrid clones encoding the ligand-binding and activator function-2 domains (LB/AF2) of ERR1 were the most abundant of twenty-three distinct hits in iterative screening. We validated the interaction by co-immunoprecipitation using HEK-293 cells expressing the yeast-2-hybrid bait and prey clones. We transfected cells with myc-tagged KIF17-T and either GFP-tagged ERR1-LB/AF2 or control GFP-empty vector (GFP-EV). One day later we performed co-immunoprecipitation analysis using anti-myc antibodies. In these experiments, GFP-ERR1-LB/AF2, but not GFP-EV, co-precipitated with myc-KIF17-T (Figure 1B). We next performed endogenous co-immunoprecipitation experiments in ER-positive MCF-7 breast cancer epithelial cells. Cell lysates were incubated with non-specific control IgG or with anti-ERR1 IgG and applied to protein-G sepharose to isolate immune complexes. After separation by SDS PAGE, immunoblot analysis confirmed that endogenous KIF17 co-immunoprecipitated with ERR1 IgG, but not non-specific IgG (Figure 1C). Co-immunoprecipitation of endogenous ERR1 and KIF17 was also detected in ER-negative MDA-MB-231 cells (not shown). This interaction may be transient, as significant KIF17 was found in non-binding and wash fractions in these assays. Alternatively, the relatively high level of unbound KIF17 may reflect limiting amounts of ERR1 relative to KIF17.

To determine if the interaction between ERR1 and KIF17 is selective, we next tested if KIF17-T interacts with other ERR family members, and if ERR1 interacts with other kinesin family members. ERR1 is one of three ERR family members: ERR1, ERR2 (ERR beta) and ERR3 (ERR gamma). ERR1 is expressed ubiquitously while ERR3 has a more restricted tissue distribution [34, 35]. ERR2 expression is limited primarily to embryonic tissues and we do not consider it further in these studies. We co-transfected HEK-293 cells with cDNAs encoding myc-KIF17-T and either GFP-ERR1 or GFP-ERR3. One day later we performed co-immunoprecipitation analysis using anti-myc antibodies. Although ERR1 and ERR3 share significant sequence identity in their LB/AF2 domains [36], GFP-ERR3 does not co-immunoprecipitate with myc-KIF17-T (Figure 1D). Moreover, in parallel experiments, we found that GFP-ERR1 does not interact with the tail domain of the kinesin-3 family motor KIF1A (myc-KIF1A-T) (Figure 1E). Consistent with this finding, GFP-ERR1 from cell lysates was pulled down by purified, recombinant GST-tagged KIF17-T and full-length KIF17 immobilized on glutathione-sepharose beads, but not by GST-KIF1A-T (not shown). Together, these data show a selective interaction between KIF17 and ERR1.

### Expression of KIF17-Tail inhibits ERR1 transcriptional activity in breast cancer cells

**Figure 2:**
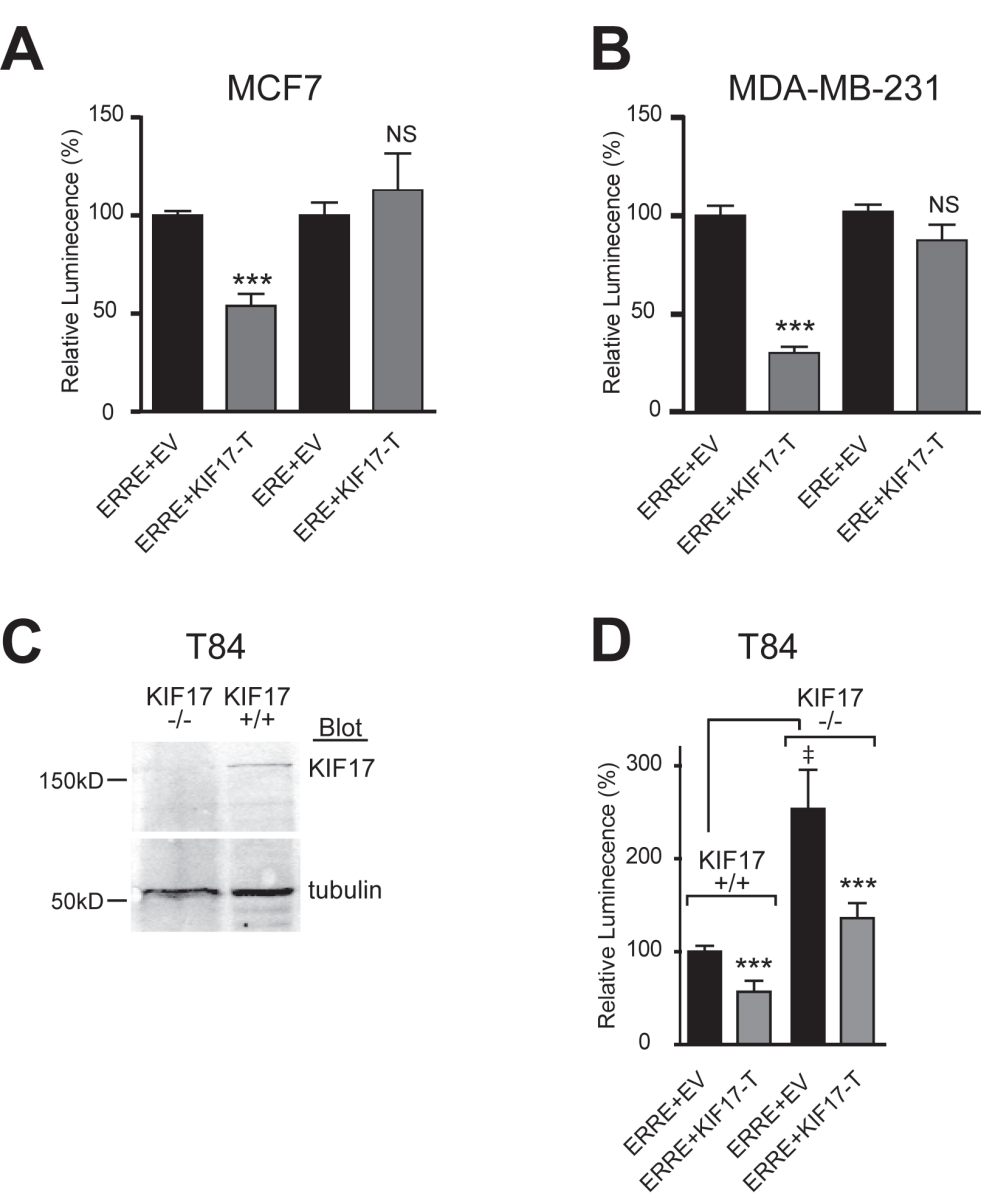
KIF17-Tail inhibits transcriptional activity of ERR1 on the ERRE, but not the ERE, in both ER-positive and ER-negative breast cancer cells Luciferase reporter assays showing transcriptional activity of endogenous ERR1 in **A.** MCF-7 and **B.** MDA-MB-231. **C.** Western blot for KIF17 in parental T84 cells (KIF17^+/+^) and T84 cells in which KIF17 was deleted by genome editing (KIF17^-/-^). **D.** Luciferase reporter assay in T84 wild type and KIF17 knock-out cells expressing GFP-KIF17-T or control GFP-EV and ERRE-Luc or ERE-Luc. Graphs show luminescence values normalized to GFP-EV. Error bars = SEM, ***p < 0.05. In panel D, GFP-EV is also normalized to parental, KIF17^+/+^ cells. Error bars = SEM, ^‡^*p* < 0.05. Data is pooled from ≥ 3 experiments performed in triplicate.

To determine if the interaction of KIF17 with ERR1 has functional significance, we tested the effects of expressing KIF17-T on ERR1 transcriptional activity in ER-positive and ER-negative cells. MCF-7 (Figure 2A), MDA-MB-231 (Figure 2B) or MCF-10a (not shown) cells were co-transfected with the ERR1 responsive element fused to luciferase (ERRE-Luc; [37]) and mCherry-tagged KIF17-T (mCh-KIF17-T) or control, empty vector (mCh-EV). Luminescence was measured 24 hours after transfection as a readout of transcriptional activity. In control cells (mCh-EV, normalized to 100%), luminescence signal was routinely robust. In cells expressing mCh-KIF17-T, we measured a significant reduction in luminescence when normalized to controls.

ERR1 and ER alpha share ~30% identity in their LBD/AF2 domains [38], and ERR1 can activate a subset of ER transcriptional targets using ER responsive elements (ERE) [4, 6–10]. Considering this, we also tested if KIF17-T interacts with and impacts ER transcriptional activity, or if it is selective for ERR1. Co-expression of KIF17-T with an ER reporter, ERE-Luc [39], had no effect on luminescence in either ER-positive (Figure 2A) or ER-negative (Figure 2B) cell lines. In addition, ER did not co-immunoprecipitate with KIF17-T (not shown). These data further show that the KIF17 tail acts on ERR1 selectively and irrespective of ER status.

The above data demonstrate effects of an overexpressed KIF17 fragment on ERR1. To determine if KIF17 plays a physiological role in regulating ERR1, we analyzed ERRE-Luc reporter activity in genome-edited, KIF17 knock-out T84 human colon epithelial cells (KIF17^-/-^, Figure 2C). Wild-type T84 cells (KIF17^+/+^) and genome edited cells were co-transfected with ERRE-Luc and mCh-EV control or mCh-KIF17-T, and luminescence was measured 24 hours later. In KIF17^-/-^ cells co-expressing mCh-EV, ERRE-Luc luminescence was elevated significantly as compared with KIF17^+/+^ cells (Figure 2D). Importantly, this increase was reversed when cells were also co-transfected with mCh-KIF17-T, demonstrating that the KIF17 tail domain can inhibit ERR1 activity in cells lacking endogenous KIF17. Together, these data suggest KIF17 acts as a repressor of ERR1 transcriptional activity.

### Expression of KIF17-Tail inhibits nuclear translocation of ERR1 in breast cancer cells

**Figure 3:**
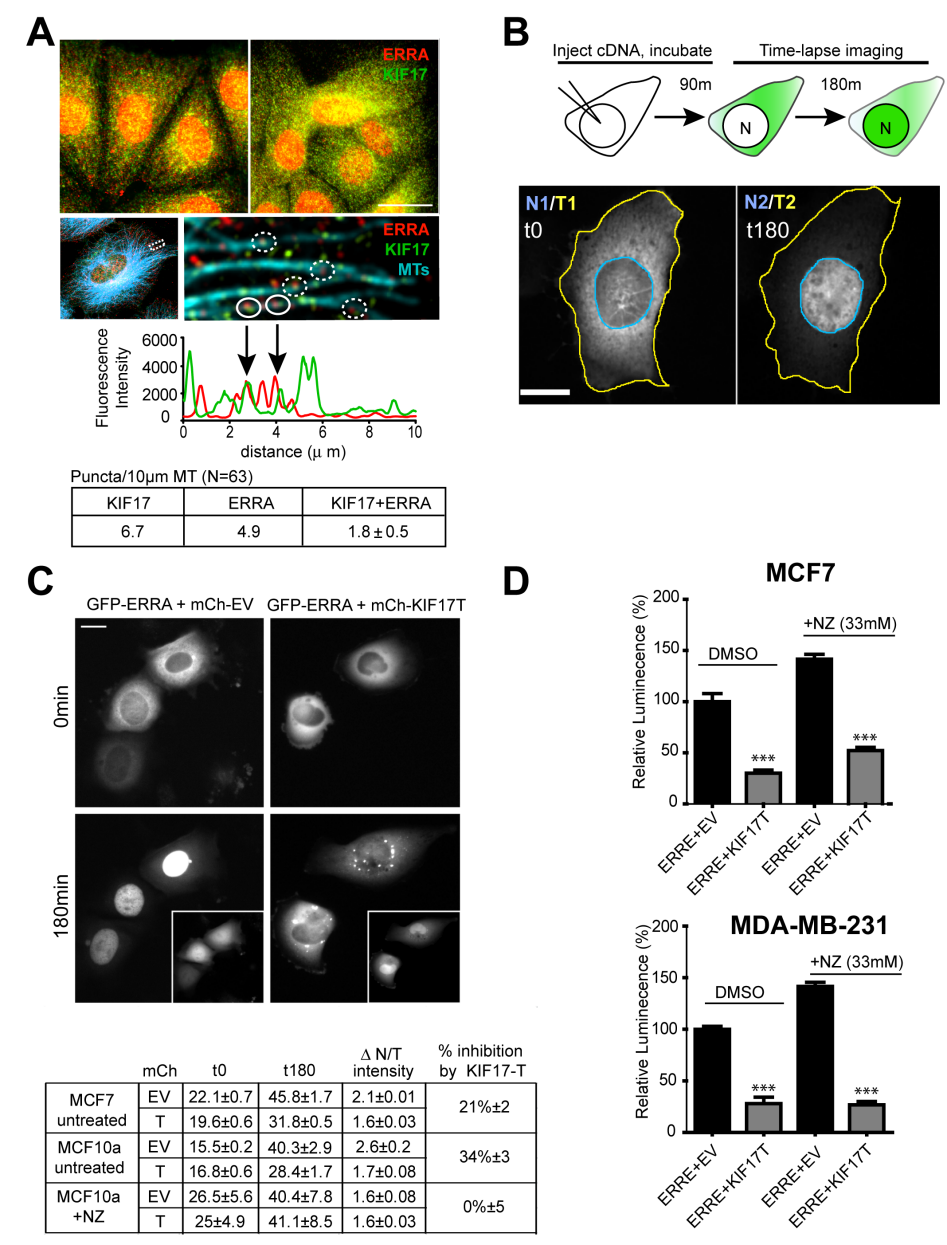
KIF17-Tail attenuates nuclear accumulation of ERR1 in both ER-positive and ER-negative breast cancer cells **A.** Upper panels: Localization of endogenous ERR1 (red) and KIF17 (green) in MCF7 (left panel) and MCF10a (right panel) cells. Lower panels: Localization of ERR1, KIF17 and MTs (cyan) in MCF-7 cells. ERR1 signal in this image was attenuated by modifying the LUT so that the MT array could be more easily visualized. The ROI indicated in this panel showing the entire cell is magnified in the right panel to highlight ERR1 and KIF17 along individual MTs. Graph and table show line-scan and quantification of ERR1 and KIF17 fluorescence intensities over a 10μm length of an individual MT. Solid circles on the image show overlapping fluorescence peaks on the line-scan. Dashed circles show other overlapping puncta on different MTs that are not represented in the line-scan. *N* = 63 MTs analyzed. **B.** Schematic describing the experimental protocol used for time-lapse imaging of GFP-ERR1 nuclear accumulation. Images show representative cells expressing GFP-ERR1 after cDNA injection in the first (t0) and last (t180) frames of a time-lapse in MCF-7 cells. After image acquisition, regions of interest were drawn defining individual cells (yellow outline) and nuclear boundaries (blue outline) at the first and last time points. GFP integrated fluorescence intensities were measured and the ratio of Nuclear/Total (N/T) GFP-ERR1 was calculated. **C.** MCF-7 cells injected with GFP-ERR1 and mCh-KIF17-T or control, mCh-EV cDNAs. Images show GFP-ERR1 in first and last frames of time-lapse recordings. Insets: mCh-EV control and mCh-KIF17-T in injected cells. Table: pooled data from 3 experiments reporting (i) % nuclear GFP-ERR1 at indicated times, (ii) the change in % nuclear fluorescence (ΔN/T), and (iii) the % inhibition of nuclear accumulation by expression of KIF17-T. **D.** Luminescence measured in untreated and NZ-treated MCF7 and MDA-MB-231 cells expressing ERRE-Luc and GFP-KIF17-T or control GFP-EV. Graphs show normalized luminescence values pooled from ≥ 3 experiments performed in triplicate. Error bars = SEM. *** *p* < 0.05. Scale bars = 25μm unless otherwise noted.

**Figure 4:**
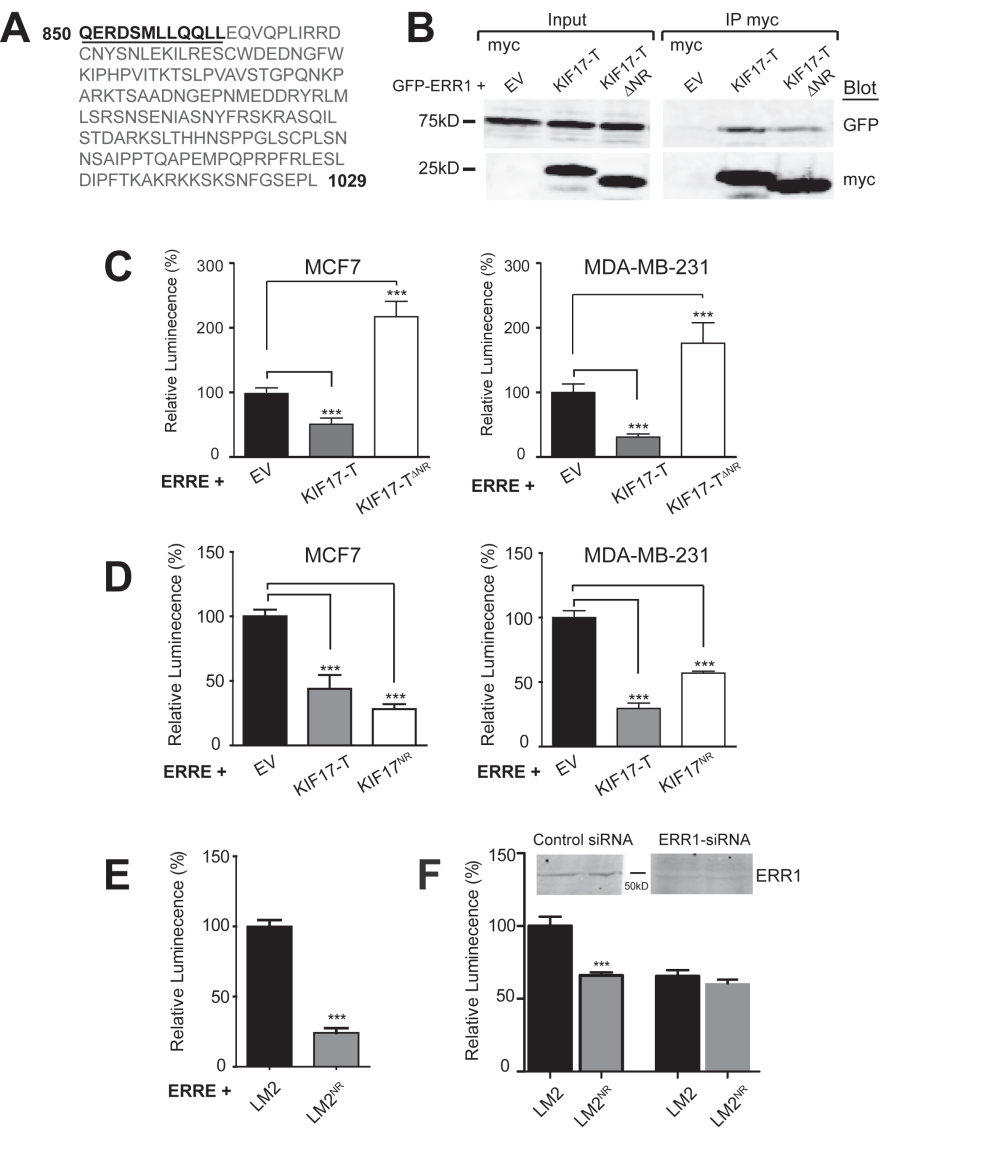
A 12 amino acid peptide in the KIF17-Tail is necessary and sufficient to inhibit ERR1 transcriptional activity **A.** Sequence of the KIF17-Tail. The 12 amino acid NR box peptide containing the LXXLL motif is underlined. **B.** Co-immunoprecipitation of GFP-ERR1 with myc-EV control, myc-KIF17-T or myc-KIF17T^ΔNR^ expressed in HEK293 cells. Immunoprecipitates and total lysates were analyzed by immunoblot using anti-GFP and anti-myc IgG. **C.**, **D.** Luciferase reporter assays showing transcriptional activity of endogenous ERR1 in MCF7 cells expressing ERRE-Luc and myc-EV, myc-KIF17-T, myc-KIF17^ΔNR^ (panel C) or myc-KIF17^NR^ (panel D). **E.** Luciferase reporter assays showing transcriptional activity of endogenous ERR1 in parental LM2 and LM2^NR^ cells transfected with ERRE-Luc. **F.** Same as in panel E, but comparing cells transfected with control or ERR1 siRNAs. Graphs show normalized luminescence values pooled from ≥ 3 experiments performed in triplicate. Error = SEM. ***p < 0.05.

Immunofluorescence analysis of endogenous ERR1 and KIF17 in ER-positive and ER-negative cells showed that ERR1 and KIF17 localized in the cytoplasm and nucleus (Figure 3A, upper panels). KIF17 also localized on MTs, as expected for a MT-associated motor and as described previously [40], and nuclear KIF17 was not unexpected as it contains a nuclear localization signal (NLS) in its C-terminal tail (see Figure 4A and [41]). Although cytoplasmic KIF17 and ERR1 puncta were numerous, we only measured a significant colocalization between the two proteins when we analyzed their distributions specifically along MTs. Line-scan analysis of ERR1 and KIF17 along MTs (Figure 3A, lower panels, graph and table) revealed that 37% of ERR1 puncta colocalized with KIF17, as compared with 18% measured after shifting the KIF17 image by 5 pixels to detect random co-distribution.

Kinesin-tail domains often have dominant-negative effects on protein translocation when overexpressed in cells, as they bind cargo but do not move on MTs [25, 42]. Because a portion of cytoplasmic ERR1 colocalized with KIF17 on MTs, we tested if expression of KIF17-T affected translocation of ERR1 from the cytoplasm to the nucleus. We expressed GFP-ERR1 in cells acutely by cDNA injection and monitored newly synthesized protein by time-lapse fluorescence microscopy (Figure 3B). ER-positive and ER-negative cells were injected with GFP-ERR1 and mCh-EV or mCh-KIF17-T cDNAs and incubated for 90 minutes to allow for protein expression. Cells were then transferred to a temperature-controlled chamber on the microscope and imaged live at 10min intervals for 180min. After image acquisition, we quantified GFP-ERR1 fluorescence in individual cells and cell nuclei and determined the ratio of nuclear:total GFP (N/T) per cell over time (Figure 3B and Table in 3C). At the start of recordings, most GFP-ERR1 localized to the cytoplasm with only ~15-25% in the nucleus in MCF-7 and MCF-10a cells (Figure 3C). In control cells co-expressing mCh-EV, ~40-50% of GFP-ERR1 localized to the nucleus 3h later. By contrast, in cells co-expressing mCh-KIF17-T, only ~30% of GFP-ERR1 localized in the nuclear compartment. Thus relative to control cells expressing GFP-EV, KIF17-T reduced nuclear GFP-ERR1 by accumulation 25-35%. Because the KIF17-tail encodes an NLS, a large fraction of mCh-KIF17-T also accumulated in nuclei (Figure 3C, insets); this likely impinges on the ability of expressed KIF17-T to maximally inhibit ERR1 nuclear translocation in these assays. Despite this limitation, our results suggest that one way the KIF17 tail affects ERR1 transcriptional function is by inhibiting its accumulation in the nucleus.

If KIF17 acts as an active transporter of ERR1 we would expect ERR1 nuclear accumulation to be dependent on an intact MT array. To test this, we treated cells with the MT depolymerizing agent nocodazole (NZ; 33μM) immediately following cDNA injection. NZ treatment accelerated the accumulation of nuclear GFP-ERR1 in all cells at early time points of the recording, but did not alter the final proportion of nuclear:total GFP-ERR1 in cells expressing either mCh-EV or mCh-KIF17-T (Figure 3C, Table). This suggests KIF17-T affects nuclear translocation of ERR1 by sequestering it on MTs and is consistent with data showing that purified KIF17-Tail can bind MTs *in vitro* [43]. Interestingly, although nuclear accumulation of ERR1 was not inhibited by MT depolymerization, expression of the KIF17-T still inhibited ERR1 transcriptional activity in luciferase reporter assays under these conditions (Figure 3D). From these data, we conclude that the KIF17 Tail domain can inhibit ERR1 function in the nuclear compartment, independent of MTs.

### A KIF17-Tail peptide containing an LXXLL NR box motif is necessary and sufficient to inhibit ERR1-mediated transcription

To more fully characterize how KIF17 interacts with ERR1 and to further explore the mechanism by which it affects ERR1 function, we analyzed the amino acid sequence of the KIF17 tail domain. We identified a nuclear receptor box (NR box) motif comprised of LXXLL residues near the N-terminal region of KIF17-T (Figure 4A). The LXXLL motif is found in nuclear receptor co-activators and mediates their binding to nuclear receptors [31–33]. To determine if this LXXLL motif is necessary for inhibition of ERR1 by KIF17-T, we generated an N-terminal truncation mutant in which 12 amino acids, inclusive of the NR box, were removed from KIF17-T (KIF17-T^ΔNR^, underlined amino acids were deleted, Figure 4A). We co-expressed GFP-KIF17-T^ΔNR^ and ERRE-Luc in ER-positive and ER-negative cells and measured luminescence 24h after transfection. Unlike wild-type KIF17-T, KIF17-T^ΔNR^ did not inhibit ERR1 transcriptional activity, and instead amplified transcription relative to controls (Figure 4C). Interestingly, we found that myc-KIF17-T^ΔNR^ co-immunoprecipitated with GFP-ERR1 (Figure 4B), albeit less efficiently, and that expression of KIF17-T^ΔNR^ attenuated ERR1 nuclear accumulation to the same extent as KIF17-T (not shown). Thus these 12 amino acids are not required for binding of KIF17-T to ERR1, but are necessary to modify ERR1activity. Considered together, these data suggest additional sequences in the KIF17-Tail domain contribute to ERR1 binding, and that the effects of KIF17 on ERR1 function are mediated primarily through interaction of the KIF17 NR box motif with ERR1 in the nuclear compartment.

To determine if this LXXLL-containing peptide in KIF17-Tail is sufficient to inhibit ERR1 function, we generated expression constructs encoding only these 12 amino acids (KIF17-T^NR^, underlined amino acids, Figure 4A). We co-expressed ERRE-Luc and GFP-KIF17-T^NR^ in ER-positive and ER-negative cells and measured luminescence 24h after transfection. KIF17-T^NR^ inhibited ERR1-mediated transcription, like wild-type KIF17-T, as compared with cells co-expressing empty vector controls (Figure 4D). Expression of myc-tagged KIF17-T^NR^ was equally effective in reducing luminescence, showing that the GFP moiety does not interfere with peptide activity (data not shown).

We also generated a cell line stably expressing GFP-KIF17-T^NR^ peptide at lower levels than in transiently transfected cells, which we use in tests of the physiological consequences of KIF17-mediated modulation of ERR1 (see below). Stable transfectants were produced in the highly invasive MDA-MB-LM2 cell line [44] and are denoted LM2^NR^ henceforth. Analysis of luciferase activity in parental LM2 and LM2^NR^ cells 24hr after transfecting ERRE-Luc showed that luminescence was significantly lower in LM2^NR^ as compared with control LM2 cells (Figure 4E). We measured a similar reduction in luciferase activity using LM2 cells stably expressing GFP-KIF17-T (not shown). Importantly, the reduction in ERRE-Luc activity measured in LM2^NR^ cells was similar in LM2 parental cells depleted of ERR1 by siRNA-mediated knock-down; ERR1 depletion in LM2^NR^ cells did not result in further reduction of luciferase activity (Figure 4F). This finding suggests that the remaining transcriptional activity measured in these cultures is likely due to the activity of ERR3, which also binds the ERRE [10] and is consistent with our finding that the KIF17-Tail does not interact with ERR3. Together, these data show that a KIF17-Tail dodeca-peptide containing an NR box motif is necessary and sufficient to inhibit ERR1-mediated transcription.

### The KIF17 NR box motif attenuates ERR1 transcriptional activity selectively and inhibits cell invasion through matrigel

**Figure 5:**
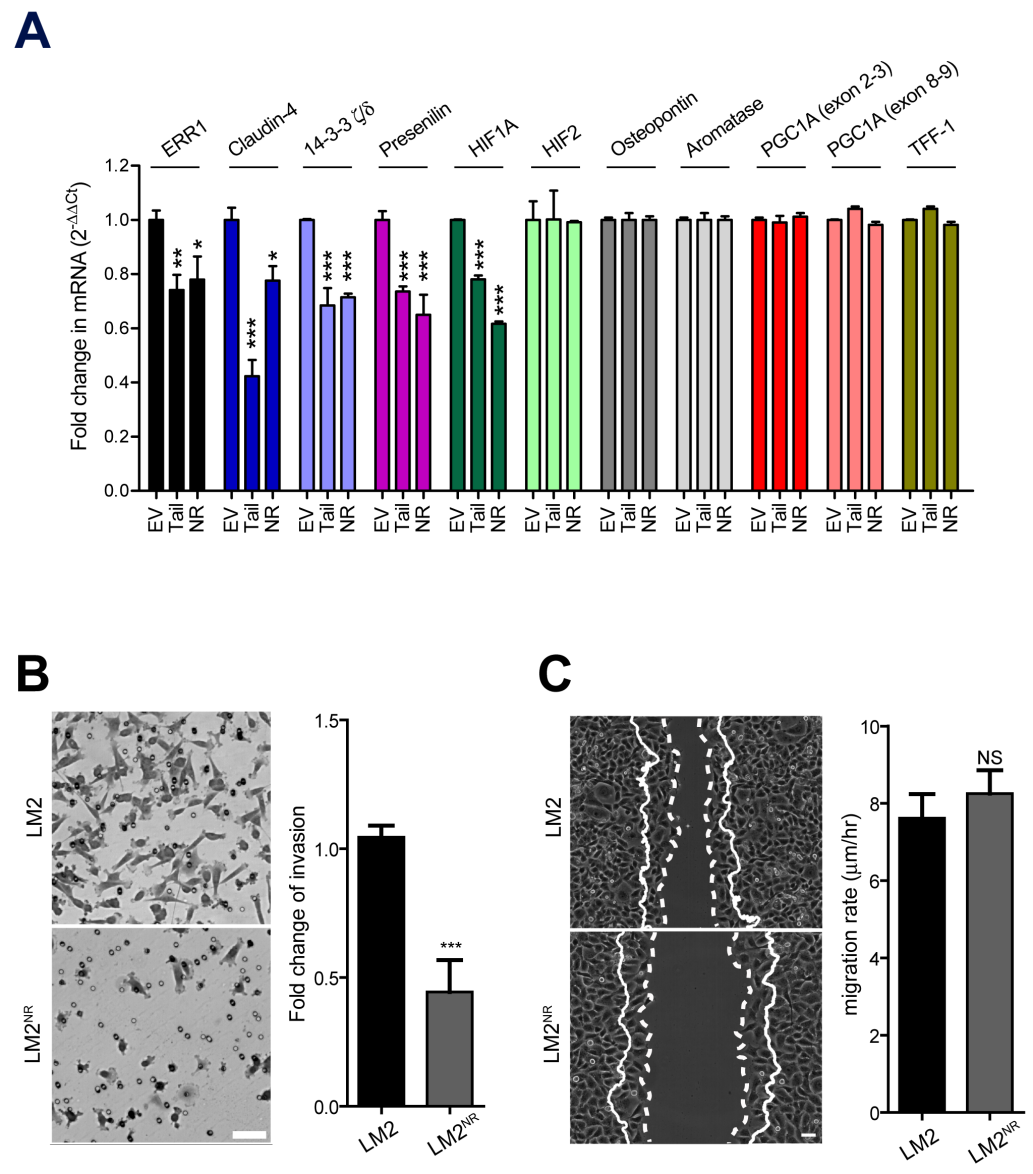
Stable expression of KIF17^NR^ or KIF17-Tail in LM2 cells attenuates transcription of a subset of ERR1 targets and inhibits invasion through materiel **A.** qPCR analysis of known and possible new ERR1 targets in LM2, LM2^NR^ and LM2-KIF17-Tail cells. Error = SEM. ***p < 0.0005, **p < 0.005, *p < 0.05. **B.** Trans-well Matrigel invasion of parental LM2 and LM2^NR^ cells. Images show representative regions of invaded cells. Graph shows fold change in invasion compared to parental LM2 cells and are pooled from duplicate samples from ≥ 3 experiments. Error bars = SEM. ***p < 0.05. Scale bar = 25μm. **C.** Cell migration into a wound by LM2 and LM2^NR^ cells. Solid lines show the cell front at the first time point. Dotted lines show the cell front in the final frame of the time-lapse (*t* = 6hrs). Scale bar = 50μm. Images are representative of cell migration in 6 random fields over time. Graph shows pooled data from ≥ 3 experiments. Error bars = SEM.

To determine if expression KIF17-T or KIF17-T^NR^ affects the transcription of known and potentially new ERR1 targets, we performed qPCR using RNA isolated from control LM2 cells stably expressing GFP-empty plasmid, LM2^NR^ and LM2-KIF17-T cells. In our analysis of previously described ERR1 targets [6], we found that stable expression of GFP-KIF17-T or GFP-KIF17^NR^ significantly reduced transcription of ERR1 and HIF1A, but not osteopontin, aromatase, PGC1A or TFF-1 (Figure 5A). As expected in the case of HIF1A suppression, a preliminary differential RNAseq analysis of LM2^NR^ and control LM2 cells (not shown) also revealed attenuated expression of ICAM-1 and angiopoietins, which are regulated by HIF1A [45]. Consistent with the lack of change in PGC1A, a key co-activator of genes involved in mitochondrial biogenesis and function, we observed no differences in mitochondrial mass or function in fluorescence-based assays (not shown), nor did we detect significant changes in mitochondrial genes by RNAseq in LM2 ^NR^ cells. Thus the KIF17-Tail domain and the KIF17 NR box motif peptide inhibit transcription of a subset of known ERR1 targets.

Stable expression of GFP-KIF17-T or GFP-KIF17^NR^ in LM2 cells also significantly inhibited transcription of Claudin-4, 14-3-3ζ/δ, and presenilin (Figure 5A), potentially new ERR1 targets we identified as down-regulated in LM2^NR^ cells by RNAseq analysis (not shown). Whether these represent *bona fide* ERR1 transcriptional targets is not yet clear and will be determined directly by ChIPseq in follow-up studies. Claudin-4 and 14-3-3δ/ζ, are of particular interest in the breast cancer landscape. High expression of claudin-4 in primary tumors, and particularly in distant metastases, is a negative prognostic indicator in ER(-) and triple-negative breast cancer patients and is a powerful predictor of survival [46–50]. High expression of 14-3-3δ/ζ is a negative prognostic biomarker associated with tamoxifen resistance in ER+ patients [51–53]. Identifying potential ways to modulate their expression could reveal additional cancer treatment modalities.

To determine the consequences of KIF17 mediated modulation of ERR1 transcriptional targets on cell behavior, we performed trans-well invasion and 2D migration assays with parental LM2 and LM2^NR^ cells. For invasion assays, serum-starved cells were seeded in serum-free medium on the upper surface of Matrigel-coated trans-well filters with 8μM pores. Complete medium containing 5% FBS was added to the lower chamber and cultures were incubated 10h before fixation and analysis of cells that had invaded the Matrigel and emerged on the bottom surface of the filter. Invasion of LM2^NR^ cells was significantly impaired as compared with parental LM2 cells (Figure 5B). These results are consistent with reduced invasion observed with siRNA mediated knock-down of ERR1 levels seen in multiple different cell types [54]. For analysis of migration in 2D, monolayer cells were scratch-wounded and cell migration/wound closure was monitored by time-lapse imaging over a 6h period. We measured no significant differences in cell migration rate and extent of wound closure between parental LM2 and LM2^NR^ cells (Figure 5C). This contrasts reports that depletion of ERR1 with siRNA inhibits both cell invasion (3D) and migration (2D) [54] and likely reflects selective effects of expressing KIF17-T^NR^ on the ability of LM2^NR^ cells to degrade extracellular matrix. Thus inhibition of ERR1 activity with the KIF17 tail NR box peptide does not globally affect cellular processes associated with downstream outputs of ERR1 transcription. This finding also highlights the potential of this peptide as a selective inhibitor of ERR1 regulated cell behaviors.

## DISCUSSION

The data presented here document a previously unreported interaction between the kinesin KIF17 and the orphan nuclear receptor ERR1 and suggest KIF17 plays a selective, regulatory role in modulating ERR1 transcriptional activity and downstream cellular behaviors associated with expression of ERR1 target genes. Our data showing that ERR1 activity is elevated significantly in KIF17^-/-^ cells, and that re-expression of the KIF17 tail domain is sufficient to reduce ERR1 activity back to normal levels measured in wild-type KIF17^+/+^ cells further indicates that this kinesin has a physiologically relevant function in modulating ERR1-mediated transcription.

**Figure 6:**
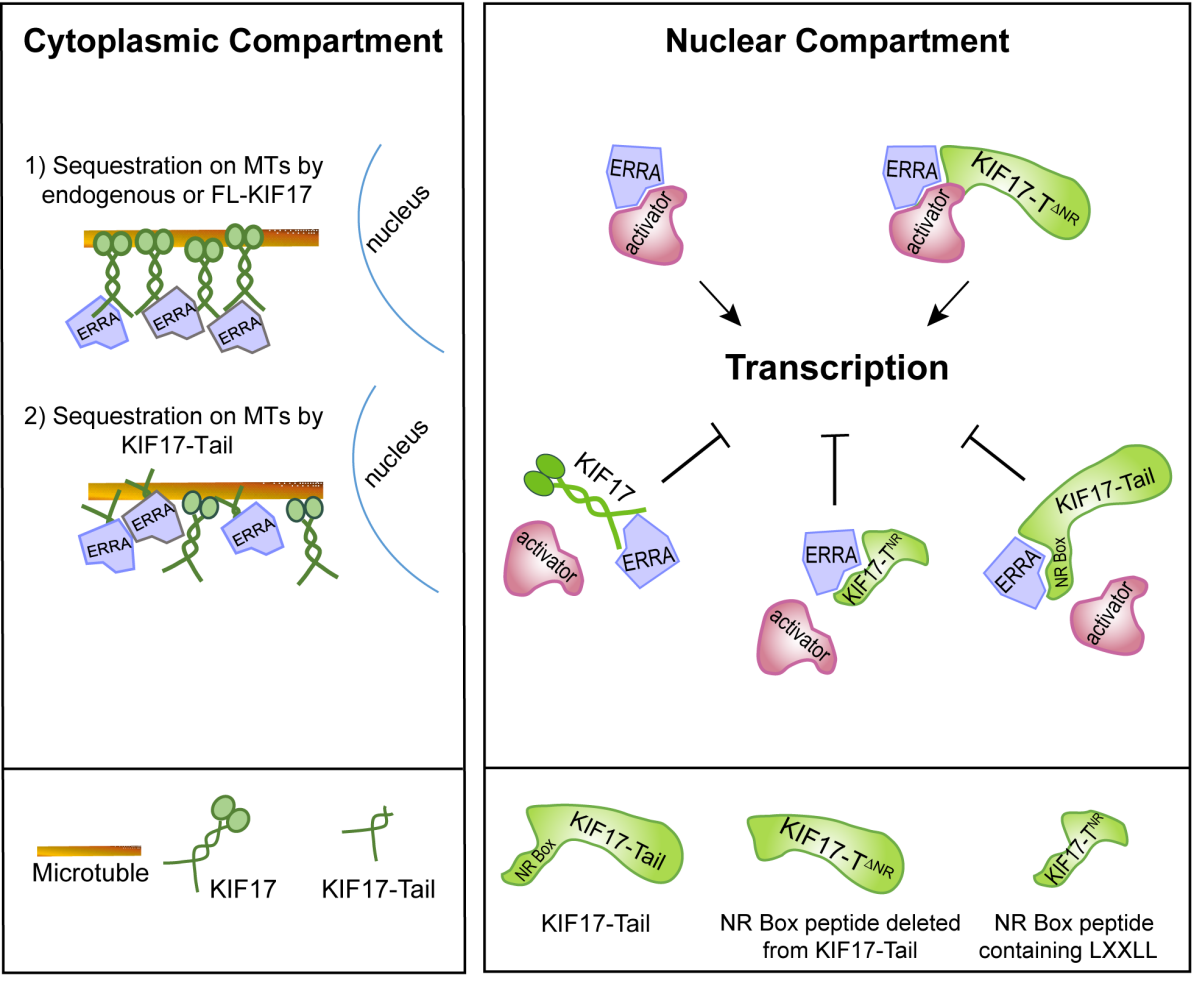
Proposed model of KIF17-mediated regulation of ERR1 Endogenous KIF17, expressed KIF17-FL, or KIF17-Tail in the cytoplasmic compartment could impact ERR1 function by sequestering ERR1 on MTs in the cytoplasm. In the nuclear compartment, endogenous KIF17, expressed KIF17-FL, KIF17-Tail or KIF17-T^NR^ could impact ERR1 function by competing with transcriptional coactivators for binding to ERR1. KIF17^ΔNR^ lacking the NR box motif binds ERR1 but does not inhibit its activity; thus the NR box is necessary for KIF17-mediated modulation of ERR1 nuclear function.

Cumulatively, our data lead us to propose a model in which KIF17 can modulate ERR1 by two possible, non-exclusive mechanisms: by controlling the nuclear-cytoplasmic distribution of ERR1 and by acting as a repressor of ERR1-mediated transcription in the nucleus (Figure 6). This idea is supported by data showing that (i) endogenous KIF17 (and expressed KIF17 or KIF17-Tail) localizes to both the cytoplasmic and nuclear compartments and (ii) over-expressed KIF17-Tail attenuates nuclear accumulation of newly synthesized ERR1 and inhibits ERR1 transcriptional activity in cells with or without an intact MT network. Several reports show that kinesins, including the testis specific isoform of KIF17 (KIF17b), can bind and sequester transcriptional regulators in the cytoplasm as a way to modify transcriptional signaling [27, 30, 55, 56]. An intact MT network is needed for the sequestration function of these kinesins, but MT-stimulated, kinesin ATPase activity is not, demonstrating that these motors participate in transcriptional regulation by acting as cytoplasmic tethers rather than active transporters. Protein tethering on MTs represents a potentially effective way to insure proper temporal and spatial control of regulated transcription pathways as MTs are the major cellular highways for long-distance transport and can facilitate productive encounters between signaling pathway molecules at discrete locations in cells. MT motors can also impact transcription by acting in their capacity as transporters, and this has been well documented in the case of cytoplasmic dynein, another class of MT-associated motor, which delivers its transcription factor cargoes to the nucleus for import [57–60]. Because we find that inhibition of ERR1 nuclear accumulation by KIF17-Tail requires intact MTs, we envision KIF17 most likely impacts ERR1 localization by acting as a MT tether. In addition, the KIF17 tail alone is not competent to move on MTs, but it can bind MTs *in vitro* independent of its canonical N-terminal, MT-binding and motor domain [43]. As such, its ability to inhibit ERR1 nuclear accumulation in our assays was not unexpected and further supports the idea that KIF17 can act in part as a cytoplasmic tethering factor for ERR1.

KIF17 could also affect ERR1 transcriptional activity by repressing its function within the nuclear compartment. This idea is bolstered by our finding that, in addition to an NLS, the KIF17 tail domain contains a conserved LXXLL NR box motif found in transcriptional co-activators. Transcriptional co-factors regulate binding of ERR1 to ERREs on target gene DNA [61, 62]. For canonical NRs, ligand binding induces a conformational change that accommodates the NR box motifs co-activators [33]. Distinctive NR box-flanking residues classify co-activators into three general categories and mediate recognition of additional NR structural features that impact their binding to specific NRs in response to diverse signaling pathways [33, 61]. Unlike canonical NRs, ERR1 and other orphans interact with co-regulators in a ligand-independent manner and thus can affect transcription constitutively [9, 11]. However, ERR1-coactivator interactions are regulated by competitive binding at co-activator sites, and by signal-mediated post-translational modifications that affect co-activator affinity for ERR1 [12, 13, 17, 22, 63–65]. Here, we show that a 12 amino acid KIF17 NR box peptide is necessary and sufficient to inhibit ERR1 activity. It is also sufficient to inhibit invasive behavior of breast cancer cells. This peptide bears signatures of NR co-activators with hydrophobic amino acids at the -1 position relative to LXXLL [31, 33]. Interestingly, deletion of the KIF17 NR box peptide does not affect binding of the KIF17-Tail to ERR1 and expression of KIF17-TΔ^NR^ results in increased ERR1 activity. This is an unexpected result and we speculate that removal of the KIF17 NR peptide allows ERR1 to access additional factors which enhance its function. Together, these data suggest KIF17 inhibits nuclear functions of ERR1 by competing with co-activators for ERR1 binding.

ER-negative breast tumors are resistant to chemotherapeutics targeting ER and estrogen production, and this resistance is partially attributable to ligand-independent activities of ERR1, loss of ER-mediated competition for regulatory cofactors, and the ability of ERR1 to stimulate ER target genes independently of ER, particularly in the breast cancer landscape [6]. The association of high ERR1 levels with poor prognoses, particularly in ER-negative/ErbB2(+) and triple-negative breast cancer, has heightened interest in targeting ERR1 and its modulators pharmacologically in breast and other steroidogenic cancers [66, 67]. Inverse agonists that interact with ERR1 have been identified and, like ERR1 silencing, they retard orthotopic tumor growth and inhibit migration of breast cancer cells in culture [16, 23, 24]. Mechanistically, these compounds interact with ERR1 in its predicted, but physiologically unused ligand binding domain, inducing a conformational change that inhibits co-activator binding, and leads ultimately to ERR1 degradation by the proteasome [20–22, 68]. To our knowledge however, none of these inhibitors are in clinical trial; most likely this is because they are predicted to induce systemic toxicity considering ERR1's control of genes in multiple, essential pathways. Attractive alternative therapeutics would modify co-factor binding selectively, and could be tailored to affect only the subset of ERR1 targets altered in the disease state. The selective effects of expressing the KIF17-Tail or KIF17 NR-box peptide on expression of known, and possibly new, ERR1 targets suggest a KIF17-based treatment modality could be exploited in such a manner.

The data presented here document a new mechanism to regulate ERR1 activity in breast cancer cells and reveal a new and direct mode by which kinesins can impact transcription. Our findings support a hypothesis that KIF17-ERR1 interactions could be exploited to selectively inhibit subsets of ERR1 targets and down-stream cellular processes. Notably, the ability the LXXLL-containing KIF17 NR box peptide to inhibit ERR1 transcriptional activity suggests it could be used, with or without additional modification, as a tool to attenuate ERR1 function in breast cancer. In the future, it will be important to determine the effects of altering LXXLL flanking sequences on transcriptional outputs of ERR1 action. It will also be interesting to determine if additional kinesins are engaged similarly to regulate ERR1 and other NRs.

## MATERIALS AND METHODS

### Cell culture, transfection, and microinjection

MCF7, MDA-MB-231, MDA-MB-LM2 (LM2) and HEK293T cells (ATCC) were cultured in DMEM (4.5 g/liter glucose) with 10% FBS and 20 mM Hepes, pH 7.2. MCF10a cells were cultured in DMEM:F12 50/50 with 5% FBS, 0.5mg/mL hydrocortisone, 100ng/mL cholera toxin and 20ng/mL EGF. MCF-7, MCF-10A, MDA-MB-231 and LM2 cells were transfected with 10μg DNA using the Amaxa^TM^ nucleofector (Lonza) as recommended. HEK293T cells were transfected using JetPrime^TM^ (Polyplus Transfection, Illkirch, France) as recommended. Microinjection was performed as described [40]. Briefly, 5-20 μg/ml cDNAs in HKCl (10 mM Hepes, 140 mM KCl, pH 7.4) were pressure injected into cell nuclei using a micromanipulator (MMO-202ND; Narishige). Cells were incubated at 37°C for 90min to allow for expression of cDNAs.

### Generation of stable cell lines

LM2^NR^ cells were generated by transfecting LM2 cells with GFP-KIF17-T^NR^ followed by sorting using the FACSAria II cell sorter (BD Biosciences). Transfected cell populations were maintained in media containing 2μg/mL of G418 during the first two rounds of cell sorting, after which G418 was omitted. Genome-edited, KIF17 knock-out T84 cells were generated by transfecting plasmids designed for genome-wide, Cas9-mediated knock-out of human *KIF17* (KN209079, OriGene). Cells were passaged once and subjected to puromycin selection (6μg/mL) for one week. Individual puromycin-resistant clones were selected using cloning rings and expanded. *KIF17* knock-out was determined in each clone by Western blot. Experiments were performed in two of the KIF17-/- clonal cell lines.

### Expression constructs

KIF17-T^ΔNR^, KIF17-T^NR^ peptide, ERR1 and ERR3 were amplified by PCR from human Caco2 cells and cloned into Gateway^TM^ expression vectors (Invitrogen). Primers: KIF17-T^ΔNR^: forward; 5’-GGG GAC AAG TTT GTA CAA AAA AGC AGG CTC CGA GCA GGT GCA GCC CCT GAT TC and reverse; GGG GAC CAC TTT GTA CAA GAA AGC TGG GTC TCA CAG AGG CTC ACT GCC AAA GTT-3’. KIF17-T^NR^ peptide: forward; 5’-GGG GAC AAG TTT GTA CAA AAA AGC AGG CTC CCA GGA GCG TGA CTC CAT GC and reverse; GGG GAC CAC TTT GTA CAA GAA AGC TGG GTC CAG GAG CTG CTG CAA GAG C-3’. ERR3: forward; 5’- GGG GAC AAG TTT GTA CAA AAA GCA GGC TCC ATG TCA AAC AAA GAT CGA C and reverse; GGG GAC CAC TTT GTA CAA GAA AGC TGG GTC GAC CTT GGC CTC CAA CAT T-3’. The KIF17-Tail and KIF1A-Tail constructs were described previously [40], [69]. ERR1: forward; 5’ - GTA CAA AAA AGC AGG CTT CTC CAG GGA GGT GGT GGG C and reverse; GTA CAA GAA AGC TGG GTC TCA GTC CAT CAT GGC CTC GAG. ERRE-Luc reporter was provided by JM Vanacker (University of Lyon, France). 3X-ERE-Luc was purchased from Addgene (Plasmid 11354). All constructs were verified by sequencing prior to use in experiments.

### Knock-down of ERRA by siRNA

5μg siRNA was transfected into cells using AMAXA electroporation and ERRA levels were monitored by western blot. Cells were harvested 3 days after transfection and used in luciferase reporter assays. ERRA siRNAs used targeted sense strand 5’ GAG CAU CCC AGG CUU CUC AUT T and antisense strand AUG AGA AGC CUG GGA UGC UCT T 3’ and have been described previously [70, 71]. Scrambled siRNA was used as a control.

### Yeast 2-hybrid

A yeast-2-hybrid library from normal colon epithelial cells (25μg) was screened using the C-terminal tail domain of KIF17 as bait as described previously [72]. 7.2 × 10^6^ clones were analyzed with 432 positives detected. Clones detected in iterative rounds of screening were used as PCR templates to prepare tagged, mammalian expression constructs for further analysis. We confirmed that clones encoding a.a. 254-423 of ERR1 interacted with KIF17 tail domain by co-immunoprecipitation in transfected HEK-293 cells.

### Immunoblotting and co-immunoprecipitation

Immunoprecipitation of expressed proteins was performed using lysates from HEK293 cells transfected with indicated constructs. One day after transfection, cells were lysed in lysis buffer (50mM HEPES, pH 7.4 containing, 150mM NaCl, 1.5mM MgCl2, 0.5mM CaCl2, 10% (v/v) glycerol, 1% (v/v) Triton-X100, 1mM PMSF, and 0.5mg/ml each of leupeptin, bestatin, pepstatin) with rocking for 30 minutes. Lysates were cleared using Protein-G sepharose beads (GE Healthcare) and incubated overnight with 6-8μg rabbit anti-GFP antibody (Novus Biologicals, NB 600-303) or mouse anti-myc antibody (Sigma M4439). Immunoprecipitation of endogenous protein was performed using MCF-7 cells. Cells were grown to confluence and lysed for 30 minutes. Lysates were cleared using Protein-G sepharose and incubated overnight with 10μg mouse anti-ERR1 antibody (Abcam ab418618). Other primary antibodies include mouse anti-GFP (Roche, 1814460), rabbit anti-myc (Cell Signaling, 2276S), goat anti-ERRα (Santa Cruz Biotechnology, sc-32971) and goat anti-KIF17 IgG (M20, Santa Cruz).

### Luciferase reporter assays

24h after transfection cells were washed with PBS and lysed using Glo-lysis buffer (Promega) for 5min. Lysates were collected and mixed 1:1 with BrightGlo Luciferase assay reagent (Promega) and luminescence measured using a Luminometer (Synergy H1, BioTek). For MT depolymerization experiments, cells were treated with 33μM nocodazole for 270 minutes before addition of Glo-lysis buffer. Results were corrected for background luminescence and plotted using GraphPad Prism 5. Statistical significance, *p* < 0.05, was determined using Bonferroni analysis.

### Immunofluorescence

Cells were fixed in −20°C methanol for 1-2 min. Microtubules were detected using YL1/2 rat-anti-tyrosinated tubulin (J. Kilmartin). KIF17 was detected with rabbit anti-KIF17 IgG (Sigma, K3638). ERR1 was detected with goat anti-ERR1 IgG (Santa Cruz, sc-32971). Fluorescently conjugated secondary antibodies were from Jackson ImmunoResearch. Images were acquired with a Neo sCMOS camera (6.45μm pixels, 560MHz, Andor Technology) on a Nikon TiE inverted microscope (Nikon Inc., Mellville, NY) using 40X (NA 1.0) or 60X (NA 1.4) plan apochromat oil immersion objectives. 14-16bit images were scaled linearly to highlight features of interest and converted to 8-bit copies for figure assembly. Devices were controlled by Elements software (Nikon Instruments). Line-scan analysis: The number of ERR1 and KIF17 puncta on MTs, and their colocalization shown in Figure 4A, was determined by line-scan analysis of fluorescence intensities along 10μm of individual MTs. Overlapping fluorescence peaks were scored as colocalized puncta.

### Time-lapse imaging and analysis

For time-lapse imaging, cells were transferred to recording medium (Hanks balanced salt solution with 20 mM Hepes, 1% FBS, 4.5 g/liter glucose, essential and nonessential amino acids) and incubated at 37°C in a thermal-controlled chamber (Harvard Apparatus) on the microscope. Time-lapse images were acquired at 10min intervals for 3h using a 20x (NA 0.75) plan apochromat objective or a 20x (NA 0.5) plan fluor, phase contrast objective and images were collected with a Neo sCMOS camera as described above.

#### Nuclear translocation of GFP-ERR1

Following image collection, background fluorescence was subtracted from 16-bit images. Regions of interest (ROIs) were drawn after morphometric thresholding around individual cells and their corresponding nuclei. Integrated florescence intensities were calculated for nuclear ROI (N) and total ROI (T) and expressed as a ratio, N/T, per individual cell over time. Average N/T per cell at the start and end time points were plotted using Graphpad Prism 5. For MT depolymerization experiments, cells were treated with 33μM nocodazole (NZ) immediately following cDNA injection and cells were maintained in NZ for the duration of the experiment.

#### Cell migration

Cells were grown on cover slips until a monolayer was formed and wounded using a sterile pipette. Time-lapse recordings were started 3h after wounding, with images acquired at 10m intervals for a duration of 6 hours. Images were thresholded to define the wound area, which was quantified using Nikon Elements software. Results were plotted Graphpad Prism 5.

### Trans-well invasion assay

Matrigel™ Invasion Chambers (Corning, part #354480) were hydrated overnight in serum-free DMEM. Cells were trypsinized, resuspended in serum-free DMEM and counted. 2×10^5^ cells were added to the upper chamber in serum-free DMEM. DMEM with 5% FBS, or serum-free DMEM as a control, was added to the lower chamber. Duplicate samples for each condition were analyzed in 3 independent experiments. Cells were incubated at 37°C for 10hrs. Following incubation, media was aspirated carefully, cells were fixed in 4% paraformaldehyde for 5 minutes and stained using 0.05% crystal violet for 5 minutes. Membranes were washed, excess Matrigel and cells remaining in the upper chamber were removed using low-lint G-tip swabs. Filters were dried, excised from the inserts and mounted onto slides for imaging. 6 × 2.3mm^2^ fields per membrane were imaged using a 10x (0.3 NA) plan fluor objective. Cells stained with crystal violet were outlined by morphometric thresholding and counted using Nikon Elements software. Unusually large cell clusters/clumps were excluded from the analysis. Results were plotted using Graphpad Prism 5.

### qRT-PCR

qRT-PCR was performed on a Taqman 7900HT using standard methods. Total RNA and cDNA was obtained from stable cell lines using Qiagen RNAeasy RNA extraction kit and cDNA generated using standard methods. GAPDH was used as a control. All primers were obtained from Integrated DNA Technologies. TET/ZEN/IBFQ was used as the dye-quencher on probes for GAPDH. 6-FAM/ZEN/IBFQ was used as the dye-quencher for all other primers. The following primers were used. ERR1: Forward 5’ CTA TGG TGT GGC ATC CTG TG 3’ and reverse TCT CCG CTT GGT GAT CTC A 3’ and, Osteopontin: Forward 5’ CCC CAC AGT AGA CAC ATA TGA TG and reverse TTC AAC TCC TCG CTT TCC AT 3’, Aromatase: Forward 5’ AGA GGA AAC ACT CAT TAT CAG CA and reverse GCC TTT CTC ATG CAT ACC GAT 3’, Presenilin: Forward 5’ TCC CTT GAC TGG CTA CCC and reverse CCA GCA CAC TGT AGA AGA TGA 3’, 14-3-3 ζ/δ:Forward 5’ GCA TGA AGT CTG TAA CTG AGC A and reverse GCA CCT TCC GTC TTT TGT TC 3’, Claudin-4: Forward 5’ CCA TAT AAC TGC TCA ACC TGT CC and reverse AGA TAA AGC CAG TCC TGA TGC 3’, GAPDH: Forward 5’ ACA TCG CTC AGA CAC CAT G and reverse TGT AGT TGA GGT CAA TGA AGG G 3’, HIF2: Forward 5’ AGC CTA TGA ATT CTA CCA TGC G and reverse CTT TGC GAG CAT CCG GTA 3’, TFF1: Forward 5’ CCA TGG AGA ACA AGG TGA TCT and reverse TGA CAC CAG GAA AAC CAC AA 3’ ’, HIF1A: Forward 5’ CTC TGA TCA TCT GAC CAA AAC TCA and reverse CAA CCC AGA CAT ATC CAC CTC 3’, PGC1A (exon 8-9): Forward 5’ GTC CTT TTC TCG ACA CAG GT and reverse GTC TGT AGT GGC TTG ACT CAT AG 3’, PGC1A (exon 1-2): Forward 5’ GAG TCT GTA TGG AGT GAC ATC G and reverse TGT CTG TAT CCA AGT CGT TCA C 3’.
